# Cryptogenic ischemic stroke and silent atrial fibrillation: What is the relationship?

**DOI:** 10.1186/s40064-016-1756-x

**Published:** 2016-02-19

**Authors:** Mustafa Karaca, D. Aytekin, T. Kırıs, A. Koskderelioglu, M. Gedizlioglu

**Affiliations:** Atatürk Eğitim Araştırma Hastanesi Kardiyoloji Bölümü, Basın Sitesi 35150 Karabağlar, Izmir, Turkey; Medikalp Heart Disease Clinic Cardiology Department, Izmir, Turkey; Izmir Bozyaka State and Research Hospital Neurology Department, Izmir, Turkey

**Keywords:** Atrial fibrillation, Cryptogenic stroke, Intraatrial conduction time, Echocardiography

## Abstract

Atrial fibrillation (AF) is responsible for up to one-third of ischemic strokes and associated with silent cerebral infarctions and transient ischemic attacks. Any method that predicts the stroke or unmasks the silent PAF would contribute to the treatment of ischemic stroke. Intraatrial conduction time (ICT) has been shown to be associated with intermittent AF. In this study, we evaluated the value of ICT detected by transthoracic echocardiography in normal population and in patients with cryptogenic stroke (CS) as a risk factor for stroke. The patients with CS and with normal left ventricular function without valvular disease are included in group 1. Patients with atypical symptoms admitted to cardiology clinics without any risk factor for cardiac disease and found to be normal constituted group 2. Age, gender, weight, height, echocardiographic parameters and ICT were compared between groups. 63 and 64 subjects were included in group 1 and 2, respectively. Two groups were similar according to age and gender. Among the parameters studied, left atrial diameter and height of the patients were significantly higher in group 1 (40 ± 2 vs 37 ± 4 mm, p < 0.001 and 167 ± 9 vs 163 ± 9 cm p = 0.027, respectively). ICT was significantly higher in group 1 (131 ± 15 vs 118 ± 13 ms, respectively, p < 0.000). According to ROC analysis, a cut point of 124 ms for ICT with a sensitivity of 74 % and specificity of 73 % in patients with CS (p < 0.001). This study show us, the measurements the ICT determined by means of echocardiography is longer in patients with CS. This simple and noninvasive technique can be applied widely and lead the clinicians to adopt the use of diagnostic and the treatment procedures.

## Background

Atrial fibrillation (AF) is responsible for up to one-third of ischemic strokes and associated with silent cerebral infarctions and transient ischemic attacks (TIA; Mohr 1988). The self-terminating and often asymptomatic nature of paroxysmal AF may lead to its underdiagnoses. We suggest that a significant proportion of the unexplained strokes or TIA can be due to undiagnosed AF (Sanna et al. 2014). Any method that predicts the stroke or unmasks the silent AF would contribute to the treatment of ischemic stroke. Intraatrial conduction time (ICT) has been shown to be associated with intermittent AF (Kinay et al. 2002). Intraatrial conduction time is commonly measured by invasive electrophysiological methods, and this measurement shows congruity with non-invasive echocardiographic calculation (Karaca et al. 2007). In this study, we aimed to investigate the value of ICT in the normal population and cryptogenic stroke (CS) patients by using the noninvasive method of transthoracic echocardiography (TTE).

## Results and discussion

Groups 1 and 2 were constituted of 63 and 64 subjects, respectively. Baseline characteristics and ICT measurement of the study population are presented in Table 1. The two groups were similar according to age and gender. Among the parameters studied, left atrial diameter and height of the patients were significantly higher in group 1 (40 ± 2 vs 37 ± 4 mm, p < 0.001 and 167 ± 9 vs 163 ± 9 cm p=0.027, respectively). ICT was significantly longer in group 1 (131 ± 16 vs 118 ± 13 ms, respectively, p < 0.000).

**Table 1:** Baseline characteristics and ICT measurement of the study population

Variables	Group 1 (n = 63)	Group 2 (n = 64)	p value
Age, years	62 ± 10	62 ± 9	NS
Male/female	38/26	39/24	NS
Height, cm	167 ± 9	163 ± 9	0.027
Weight, kg	75 ± 1	76 ± 1	NS
LA dimension	40 ± 2	37 ± 4	<0.001
EF	57.2	58.5	NS
ICT	131 ± 15	118 ± 13	<0.000

*EF* ejection fraction, *ICT* intraatrial conduction time, *LA* left atrium

**Fig. 1:**
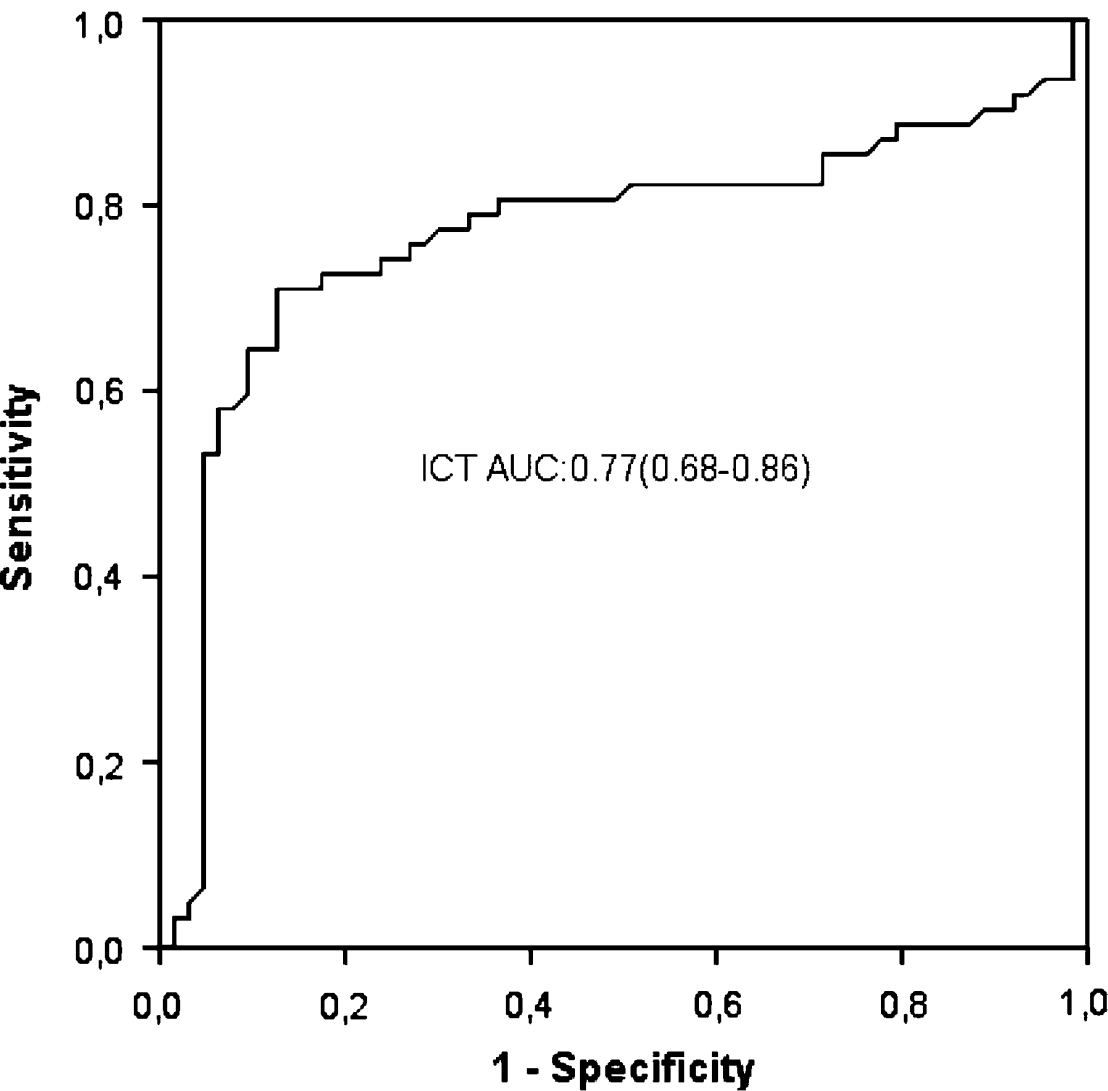
Receiver operating curve of intraatrial conduction time for cryptogenic stroke

In the present study, we found that ICT was prolonged in patients with CS compared with healthy subjects (group 1 vs group 2; 131 ± 15 vs 118 ± 13 ms, p < 0.000).According to ROC analysis, a cut point of 124 ms for ICT identified the patients at risk of CS with a sensitivity of 74 % and specificity of 73 % (p < 0.001, Fig. 1).

Cryptogenic stroke has a devastating clinical outcome. Its cause is unknown even after vigorous neurological, radiological and other laboratory evaluation. It is well established that the presence of AF is associated with an almost fivefold increased risk of stroke (Wolf et al. 1991). Silent AF and other cardiac sources may be partly responsible for CS and yet still difficult to disclose. Other risk factors related to stroke and silent AF may be value for the approach to this patient population.

The prolongation of ICT and inhomogeneous propagation of sinus impulses are well known electrophysiological characteristics in patients with AF (Villani et al. 1996; Dogan et al. 2012). Subclinical atrial dilatation may be the cause and substrate of decremental conduction properties which may lead to inducible episodes of AF (Cozma et al. 2006).

Intraatrial conduction time can be determined invasively in the EP lab. A previous study by our group has shown that there was a significant correlation between ICT measurement determined by transesophageal echocardiography and invasive electrophysiological study (Karaca et al. 2007). A limited number of studies have revealed increased episodes of AF in patients with prolonged ICT. Another study has reported that this method predicted AF in patients with a long ICT detected during open heart surgery following coronary by pass operation (Karaca et al. 2012). TEE was, however, found to be a useful tool for measuring ICT. There is a significant association between ICT and recurrence of AF (Kinay et al. 2002). In a previous study, investigators simultaneously measured the time interval between the electrocardiographic P wave and the mitral “a” wave using transthoracic Doppler echocardiography, and they compared this noninvasive ICT measurement with the invasive method (Fuenmayor et al. 2002). They concluded that transthoracic Doppler echocardiography combined with surface electrocardiography could be used for measuring the ICT with a similar accuracy as other more invasive methods. Technical improvements made this measurement more accurate and feasible.

Our study revealed that ICT detected by echocardiography was prolonged in patients with CS when compared to the normal population. Left atrial size also increased in group 1. Although this study was not designed to detect silent AF episodes in the groups, increased conduction time and left atrial enlargement are known as AF triggers. One can only speculate that group 1 patients may have increased episodes of AF. This study shows that ICT calculated by echocardiography was prolonged in patients with CS. One electrophysiological study showed the increased atrial vulnerability in young stroke patients with atrial septal abnormalities (Berthet et al. 2000).

Silent AF episodes can easily be detected in patients with implanted devices either directly from interrogation of the device or via remote monitoring (Lima et al. 2015; Healey et al. 2013). The CRYSTAL AF study showed that AF was more frequently detected with insertable cardiac monitors than with conventional follow-up in patients with a recent CS. In that study, only age and prolonged PR interval at enrollment were associated with AF. Intrinsic PR prolongation has been consistently demonstrated to be associated with AF as it represents intrinsic atrial conduction disease (Sanna et al. 2014). In patients without a cardiac device, it is very difficult to detect AF episodes. Our study results may reflect a practical approach to overcome this difficulty.

In a recent study, the EMBRACE trial, premature atrial beats were found to be associated with silent episodes of AF in Holter recordings in patients with cryptogenic emboli (Gladstone et al. 2015). Prolonged ICT may be associated with left atrial mechanical remodeling and dysfunction, both of which may be substrates for thromboembolism in CS patients. Whether increased atrial premature beats, p wave dispersion or ICT increase the tendency to tromboemboli by possibly increased AF episodes or by some unknown subclinical atrial pathology unrelated with AF is yet to be demonstrated. Since there is no clinical data supporting the use of anticoagulation regarding the prolonged ICT, indication for anticoagulation should be based on current recommendations (Glotzer and Ziegler 2013).

## Limitations

The number of subjects was too small to generalize the results. Although this study was not designed to reveal the relationship between PAF and ICT, it shows an association between ICT and stroke.

## Conclusions

This study shows that ICT measured by echocardiography was prolonged in patients with CS. This technique can be applied widely and may lead clinicians to insist on monitoring patients to reveal silent AF. This is especially noteworthy if the presence of AF would likely change the current therapeutic approach and provide additional diagnostic tools for these particular patients.

## Methods

A total of 285 consecutive patients diagnosed with cerebrovascular accident in our neurology clinic between April 2014 and January 2015 were investigated for possible CS. All patients were prospectively evaluated and regularly underwent clinical and instrumental evaluation. Patients had routine blood tests (i.e. full blood count, clotting, C-reactive protein, erythrocyte sedimentation rate, liver function, renal function, thyroid function, electrolytes, and lipid profile) after the stroke episode. Thrombophilia screening, vasculitis screening and genetic tests, where appropriate, were performed for patients with CS or those younger than 45. We did not enroll 142 patients with stroke in whom the cause of stroke was identified initially. The remaining 143 patients with possible CS were considered for evaluation.

Of the 143 patients recruited for possible CS, 63 stroke/TIA patients (38 males; mean age 62 ± 10 years) who were considered to have cryptogenic stroke after extensive radiological and cardiac investigations constituted group 1. Eighty patients who had exclusion criteria were not included in the study. All cases were reviewed by two senior neurologists and stroke was classified according to the TOAST classification (Adams et al. 1993). The diagnosis of an ischaemic stroke was based on symptoms and signs of a sudden onset focal neurological deficit, lasting at least 24 h, with corresponding findings on computerized tomography, magnetic resonance imaging scans. TIA was defined as a focal neurological deficit which resolved completely within 24 h without positive neurovascular imaging. We excluded patients with prior cerebrovascular accident, malignant arterial hypertension, uncontrolled diabetes mellitus, untreated hyperthyroidism, myocardial infarction or coronary bypass grafting history 1 month prior to stroke, valvular disease, documented history of AF or atrial flutter or presence of PFO. Patients with more than 3 days follow up in intensive care unit or requiring intubation due to respiratory failure were also excluded from the study.

Sixty-four patients (39 males; mean age 62 ± 9 years) with atypical symptoms admitted to cardiology clinics without any risk factor for cardiac disease and they were considered as healthy controls. Accordingly, they constituted group 2.

**Fig. 2:**
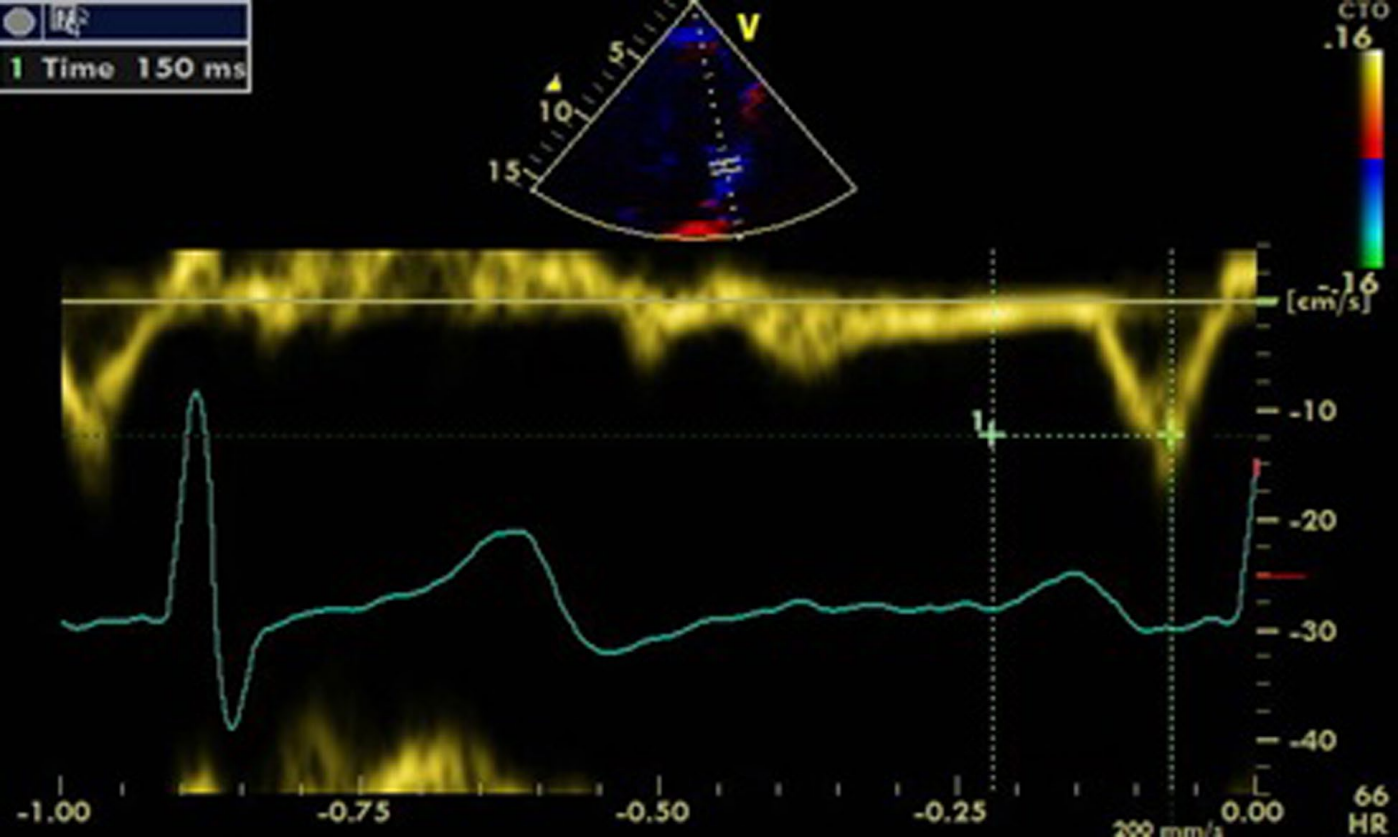
Intraatrial conduction time is defined as the time between the beginning of the p wave in surface ECG and the top of the A wave recorded from the tissue Doppler imaging from the left atrial free wall next to the mitral annulus

The echocardiography machine in the study provided real-time, simultaneous display of ECG and doppler echocardiogram of patients. Baseline echocardiography measurements recorded and compare with NORRE study (Kou et al. 2014). After the routine echocardiography evaluation, ICT was measured. The beginning of the P wave was marked in modified lead DI, in which the right arm electrode was attached on the top right of the manubrium and the left arm electrode in the fifth intercostal space at the right parasternal line (Lewis lead). The last atrial activity was marked as the top of the A wave trace which was obtained from the left atrium free wall next to the mitral annulus with tissue Doppler recording (Fig. 2).

This study was approved by our hospital review board and the informed consent was obtained from each patient.

Statistical analysis; age, gender, weight, height, echocardiographic parameters and ICT were compared between groups. Continuous variables are presented as mean ± SD, whereas dichotomous variables are described as numbers and percentages. The differences among the two groups were compared using the Chi-square test for categorical variables and Student’s t tests or Mann Whitney U test for continuous variables. A cut point of ICT for predicting of CS was calculated by using receiver operating curve (ROC) analysis, and its sensitivity and specificity were estimated. Statistical analysis was performed by using the Statistical Package for Social Sciences, version 16 (SPSS Inc., Chicago, IL, USA). A p value <0.05 was considered to indicate statistical significance.

## Abbreviations

AF: atrial fibrillation
CS: cryptogenic stroke
TIA: transient ischemic attacks
ICT: intraatrial conduction time
TTE: transthoracic echocardiography
ROC: receiver operating curve
